# Design of All-Optical D Flip Flop Memory Unit Based on Photonic Crystal

**DOI:** 10.3390/nano14161321

**Published:** 2024-08-06

**Authors:** Yonatan Pugachov, Moria Gulitski, Dror Malka

**Affiliations:** Faculty of Engineering, Holon Institute of Technology (HIT), Holon 5810201, Israel

**Keywords:** flip flop, photonic crystal ring resonator, plane wave expansion, finite-difference time domain

## Abstract

This paper proposes a unique configuration for an all-optical D Flip Flop (D-FF) utilizing a quasi-square ring resonator (RR) and T-Splitter, as well as NOT and OR logic gates within a 2-dimensional square lattice photonic crystal (PC) structure. The components realizing the all-optical D-FF comprise of optical waveguides in a 2D square lattice PC of 45 × 23 silicon (Si) rods in a silica (SiO_2_) substrate. The utilization of these specific materials has facilitated the fabrication process of the design, diverging from alternative approaches that employ an air substrate, a method inherently unattainable in fabrication. The configuration underwent examination and simulation utilizing both plane-wave expansion (PWE) and finite-difference time-domain (FDTD) methodologies. The simulation outcomes demonstrate that the designed waveguides and RR effectively execute the operational principles of the D-FF by guiding light as intended. The suggested configuration holds promise as a logic block within all-optical arithmetic logic units (ALUs) designed for digital computing optical circuits. The design underwent optimization for operation within the C-band spectrum, particularly at 1550 nm. The outcomes reveal a distinct differentiation between logic states ‘1’ and ‘0’, enhancing robust decision-making on the receiver side and minimizing logic errors in the photonic decision circuit. The D-FF displays a contrast ratio (CR) of 4.77 dB, a stabilization time of 0.66 psec, and a footprint of 21 μm × 12 μm.

## 1. Introduction

The engineering industry can be split into two groups, people who believe Moore’s law is dead and people who disagree. In 1965, Gordon Moore predicted a doubling of components per integrated circuit (IC) every year. Today, we have already reached the minimum size of a transistor that is feasible for fabrication in terms of operation and economic viability; thus, an alternative technology is needed to overcome the physical barriers of the current CMOS standard-built chips and ICs.

In the relentless pursuit of faster and more energy-efficient computing systems, the integration of photonics in digital architectures has emerged as a promising avenue [1,2,3,4,5]. Photonic-crystal (PC)-based structures offer unprecedented opportunities for realizing high-speed and low-power computing components, revolutionizing the landscape of optical computing, chips, and ICs. This paper presents a novel configuration for an all-optical D Flip Flop (D-FF) leveraging the principles of PCs.

At the heart of any digital computing system lie arithmetic logic units (ALUs) and memory components, which are pivotal for executing computational tasks and storing data. Traditional electronic ALUs and memory units are plagued by limitations such as speed constraints and susceptibility to electromagnetic interference. However, by harnessing the unique properties of light, PC-based ALUs and memory components [6,7,8,9,10] offer the promise of ultrafast operation and enhanced reliability, setting the stage for transformative advancements in optical computing. Several unique properties of light are utilized in PC-based components. Firstly, its speed is harnessed as light travels at incredibly high speeds, facilitating rapid data transmission and processing compared to traditional electronic signals. Additionally, photonic signals exhibit non-interference, making them less susceptible to electromagnetic interference and resulting in more reliable data transmission and storage, particularly in densely packed computing systems. Moreover, photonic devices typically operate at lower power levels than their electronic counterparts, leading to reduced energy consumption and heat dissipation, thereby enhancing overall system efficiency.

FFs represent essential building blocks in sequential logic circuits, facilitating the storage and synchronization of data. Conventional electronic FFs, while widely used, suffer from inherent limitations in speed and power consumption. In contrast, optical FFs [11,12,13,14] present a paradigm shift, offering ultrafast switching speeds and minimal energy dissipation. In particular, the D-FF holds significance for its simplicity and versatility in digital circuit design, making it an ideal candidate for optical implementation.

PCs, characterized by their periodic arrangement of dielectric materials, offer unparalleled control over the propagation of light at the nanoscale. By engineering photonic bandgaps and exploiting light–matter interactions, researchers can tailor the optical properties of these structures to realize efficient optical components, for instance, demultiplexers [15,16,17], logic gates [18,19], polarizers [20,21], beam combiners [22,23,24], and sensors [25,26]. This paper explores the design and optimization of PC-based components, including waveguides, ring resonators (RRs), and logic gates, to enable the realization of an all-optical D-FF.

Numerous alternative designs for PC-based D-FFs have been proposed. For example, one design integrates a distinctive arrangement of line waveguide and RR structures utilizing silicon (Si) and chalcogenide glass [27]. Another configuration for an all-optical D-FF involves employing multi-mode interference on a PC platform with a square lattice structure [28]. In our investigation, we assess the performance of our proposed D-FF structure by comparing it to these recent research findings.

To facilitate the design and simulation of complex photonic structures, advanced computational techniques such as finite-difference time-domain (FDTD) [29] and plane-wave expansion (PWE) [30] methods are employed. These numerical approaches enable the accurate modeling and analysis of light propagation within PCs, guiding the optimization of device performance and functionality.

In the proposed configuration, a quasi-square RR [31,32,33], NOT logic gate [34,35], and an OR logic gate [36,37] are utilized to realize the essential functionalities required for the implementation of the D-FF. By integrating these key components within a PC framework, the paper aims to demonstrate an all-optical D-FF that has the capability to function across all eight possible logic states. This achievement distinguishes our proposed structure from existing works, which have predominantly displayed limited logic state compatibility.

Furthermore, the use of SiO_2_ as a substrate and silicon rods in our design offers practical advantages in terms of fabrication and physical testing, as compared to conventional air-based structures prevalent in the existing literature. Beyond its immediate applications, our innovative design also holds considerable potential as a memory component for quantum computing systems.

## 2. Principle and Analysis

### 2.1. Light Interference

An important principle of the correct operation of PC technology is light interference, which is a phenomenon that occurs when two or more light waves overlap and interact with each other. One outcome of this interaction is constructive interference, which occurs when the phase difference between the waves is an even multiple of π (180°); the other outcome is destructive interference, which occurs when the difference is an odd multiple of π. The amplitude or intensity of the waves inserted into the proposed device is normalized (N.I) and measured in arbitrary units [a.u] of measurement.

### 2.2. Light Propagation

The FDTD method computationally models light propagation within waveguide structures. It discretizes space into a grid and applies Maxwell’s equations to calculate electric and magnetic fields at each point. The waveguide’s material properties and geometry are represented within this grid. An excitation source initiates light propagation through the waveguide. The FDTD algorithm iteratively updates the fields over time, simulating the evolution of electromagnetic waves within the structure. The analysis of the simulation results provides insights into light guidance and manipulation in waveguides, aiding in the optimization of optical device designs. In this research, the FDTD method was implemented using the RSoft CAD 7.1 program.

### 2.3. Photonic Band Gap

The PWE method [30] is employed to analyze photonic band gaps (PBGs) and identify operational wavelengths for photonic devices. Initially, the device structure is discretized into a periodic lattice. Each unit cell is characterized by its refractive index distribution. Plane waves are then introduced into the structure at various angles and wavelengths. Through numerical calculations, the PWE method determines the dispersion relation, which illustrates how the propagation of light waves is affected by the periodicity and refractive index distribution of the device. PBGs, regions where certain wavelengths of light cannot propagate through the structure, are identified in the dispersion relation. By examining these band gaps, one can ascertain the wavelengths at which the device will effectively function. In this context, it is important to consider the polarization modes of the electromagnetic waves, namely, Transverse Electric (TE) and Transverse Magnetic (TM) modes. The TE mode has the electric field oriented perpendicular to the direction of propagation, while the TM mode has the magnetic field oriented perpendicular to the direction of propagation. The PBG for TE and TM modes can differ, so analyzing both is crucial for a comprehensive understanding of the PC’s optical properties. The PWE method aids in the design and optimization of photonic devices by providing insights into their wavelength-selective behavior and potential performance limitations.

### 2.4. D-FF

**Figure 1:**
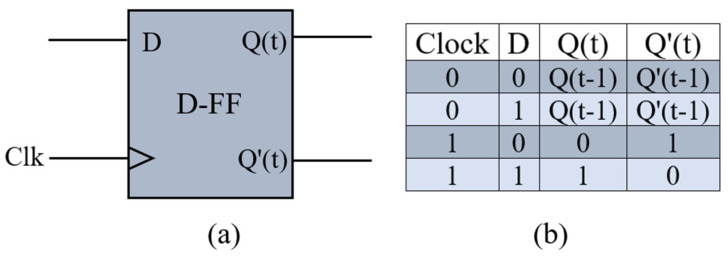
Block diagram (**a**) and accuracy table (**b**) of a D-FF.

The D-FF, commonly known as data FF, is widely used in the world of computing. It captures the input of D at a certain time in the clock cycle, such as the moment when the clock toggles from its negative edge to its positive edge, and the captured data at input D become the output Q. At all other times, the output Q stays unchanged. Output Q′ is the complement of output Q, and t refers to the current state’s time, while t − 1 refers to the previous state’s time. The block diagram of a classic D-FF is shown in Figure 1a, and the truth table of the D-FF is shown in Figure 1b.

**Figure 2:**
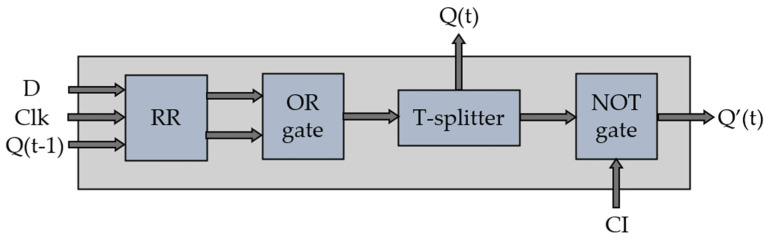
Block diagram of proposed D-FF.

The block diagram of the optical D-FF, depicted in Figure 2, relies on a quasi-square RR for amplifying and guiding the light wave. Additionally, it incorporates a T-splitter to efficiently divide the wave with minimal power loss, along with OR and NOT logic gates.

## 3. Structure

**Figure 3:**
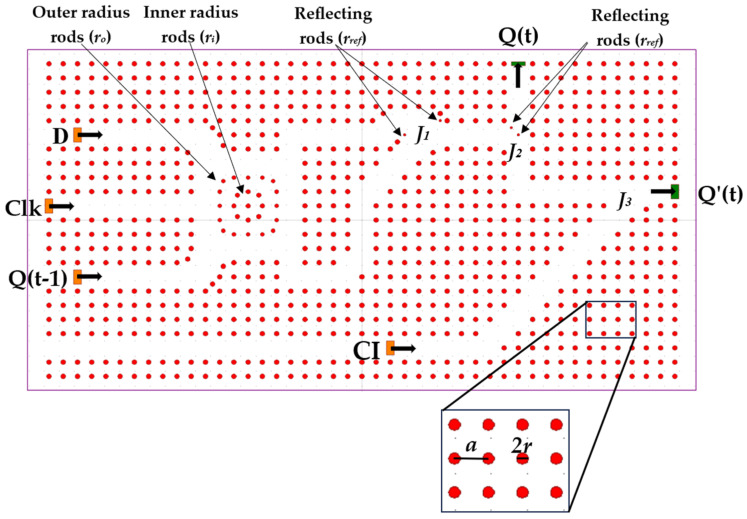
Schematic structure of the proposed all-optical D-FF.

Figure 3 illustrates the suggested clocked D-FF, which features a configuration with a square lattice layout in the X–Z plane, consisting of 45 × 23 Si rods within a SiO_2_ substrate. In the figure, the areas in red and white correspond to Si and SiO_2_, respectively. The lattice has a constant ‘*a*’ of 474 nm, with refractive indexes of 3.46 for Si and 1.45 for SiO_2_. The Si rods have a radius of 0.17 times the lattice constant ‘*a*’. These parameters affect the light confinement and transmission losses throughout the structure, they were determined as a result of optimization with an operating wavelength of 1550 nm.

The structure has four inputs: clock (Clk), D, Q(t − 1), and control input (CI); and two outputs: Q(t) and Q′(t). Input D is the data input, when the Clk input is at logic ‘1’ and output Q(t) obtains the value of D. Output Q′(t) obtains the complementary value of D (D′) when the Clk input is at logic ‘0’, output Q(t) obtains the value of input Q(t − 1), which is the previous state of Q(t), and Q′(t) obtains the complementary value of Q(t). Input CI is used to realize the NOT gate at output Q′(t). Inputs D, Clk, and Q(t − 1) are each guided by a waveguide into the RR, where the light waves either construct or destruct with each other, depending on the input logic state. The RR consists of an inner ring made of Si rods with a radius r_i_ of 0.16a and an outer ring made of Si rods with a radius ‘*r_o_*’ of 0.14a. The two outputs of the RR go into the first junction *J*_1_, which acts as an OR gate; the combined light wave travels to junction *J*_2_, which acts as a T-splitter, splitting the wave with minimal power loss to output Q(t) and junction *J*_3_. The wave encounters the wave from input CI. They destructively interfere with each other, creating a NOT gate, and the resulting wave travels to output Q′(t).

**Figure 4:**
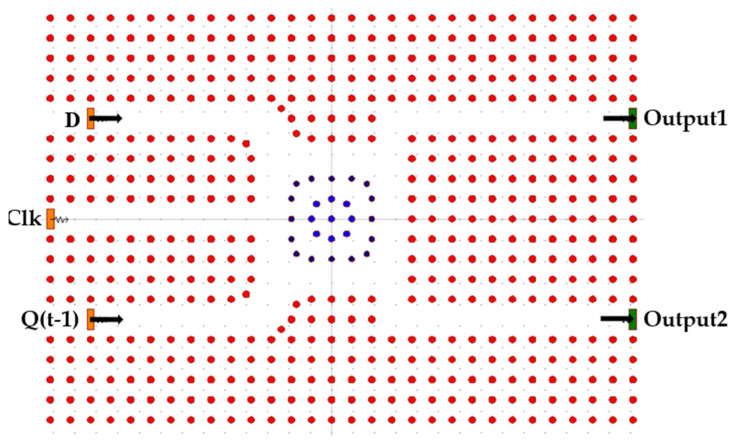
Schematic structure of RR.

To simplify and explain the operational principles of the D-FF, the structure is partitioned into distinct components. Figure 4 illustrates the first component, an RR with three inputs: D, Clk, and Q(t − 1), and two outputs.

The inner and outer radii of the ring resonator shapes were designed with inspiration from already proven similar work [38].

Since there are three inputs, there is a total of eight logic states that are addressed in Table 1. Each logic state is represented by power and phase shift; at the input D, logic ‘0’ and ‘1’ are defined as 0.1 a.u and 0.9 a.u, respectively, with a phase shift of 0. At the input Q(t − 1), logic ‘0’ and ‘1’ are defined as 0.1 a.u and 0.9 a.u, respectively, with a phase shift of π. At the input Clk, logic ‘0’ is defined as 0.9 a.u and a phase shift of π, and logic ‘1’ is defined as 0.9 a.u and a phase shift of 0.

The contrast ratio (*CR*) refers to the difference in optical power levels between the ON and OFF states, indicating the clarity and distinction between logic ‘1’ and ‘0’ outputs and is given as:

(1) $$CR=10\log_{10}\frac{P_{1}}{P_{0}}$$

where *P*_1_ and *P*_0_ represent the output power levels corresponding to logic ‘1’ and logic ‘0’, respectively.

The CR calculated at Output1 and Output2 are 3.68 dB and 3.27 dB, respectively.

**Figure 5:**
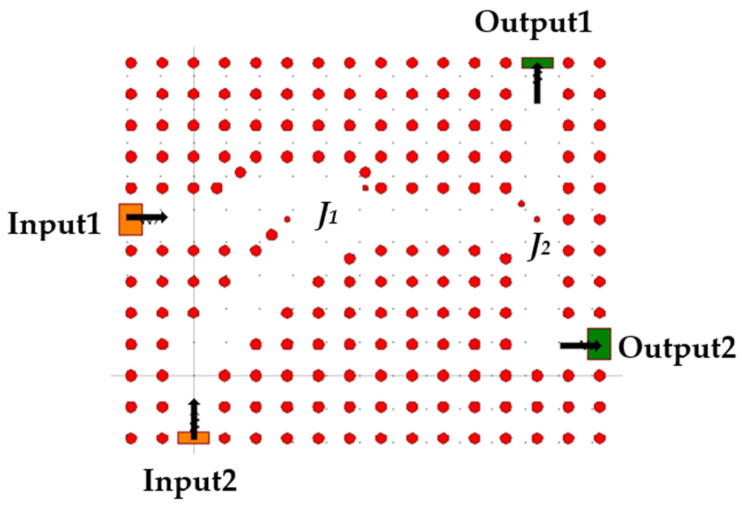
Schematic structure of T-splitter and OR logic gate.

The second component described in Figure 5 has two inputs and two outputs. The two inputs are connected to the outputs of the RR. The component realizes an OR logic gate to combine the outputs of the RR into one waveguide, and a T-splitter, to split the light wave into two waveguides, while Output1 will later be the final output Q(t), with minimal losses and the same logic state. The light waves from the inputs travel by waveguides to junction *J*_1_, where they constructively interfere; the resulting wave travels to junction *J*_2_ where it is split into two.

Since there are two inputs, there are a total of four logic states that are described in Table 2. Logic state ‘0’ is represented by a power of 0.1 a.u, and logic state ‘1’ is represented by a power of 0.9 a.u.

**Figure 6:**
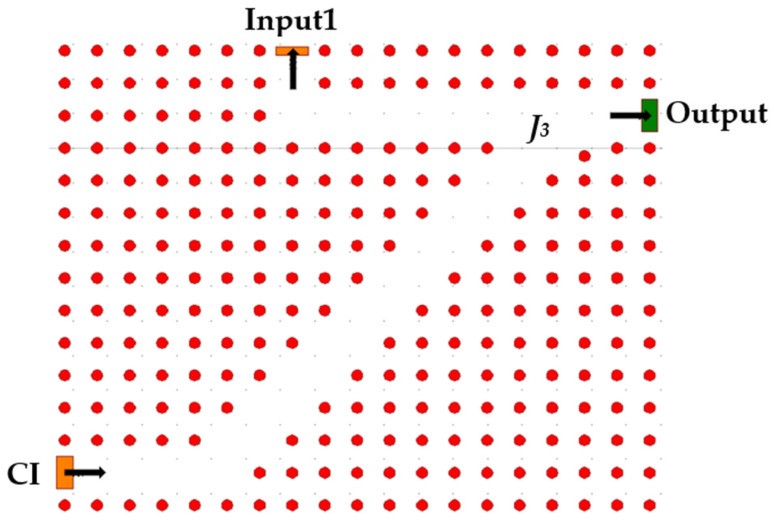
Schematic structure of NOT gate.

The third component described in Figure 6 has two inputs and one output. One of the inputs is the output of the second component, and the other is a controlled input called CI. The component realizes a NOT logic gate. The CI input is, as implied, always active, and the other input is the data input. The light waves travel by waveguides to junction *J*_3_ where they destructively interfere. The output will later become the final output Q′(t).

Since there are two inputs, there are a total of four logic states that are described in Table 3. Logic state ‘0’ is represented by a power of 0.1 a.u, and logic state ‘1’ is represented by a power of 0.9 a.u.

## 4. Optimizations

Optimizations were performed on every parameter influencing and modifying the behavior of the proposed D-FF structure. This module takes in a parameter, generates a test vector, and subsequently simulates the structure using the FDTD method.

**Figure 7:**
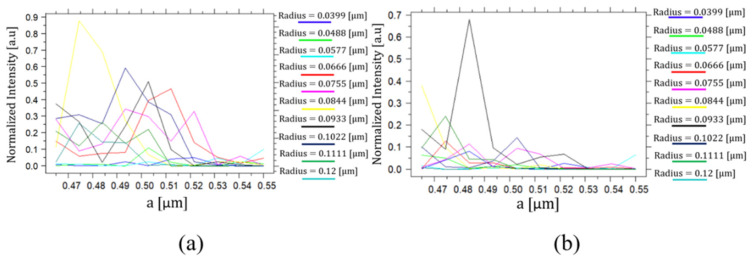
Normalized intensity at outputs (**a**) Q(t) and (**b**) Q′(t) as a function of ‘*a*’ and ‘*r*’.

The proposed structure is based on two main parameters: the lattice constant ‘*a*’ and the radius of the Si rods ‘*r*’. Optimization was made on both to find the structure configuration with optimal light confinement and minimal transmission losses. For a working wavelength of 1550 nm, Si rods in SiO_2_ substrate were used with refractive indexes of 3.46 and 1.45, respectively. Figure 7a,b shows the normalized intensity at outputs Q(t) and Q′(t), when ‘*a*’ is tested in the range of 0.465 and 0.55 μm, and ‘*r*’ is tested in the range of 0.0399 and 0.12 μm.

All optimizations performed on the proposed D-FF structure used an input state such that D, Clk, and Q(t − 1) obtain the logic values of ‘0’, ‘0’, and ‘1’, respectively. The expected outputs Q(t) and Q′(t) for this state are ‘1’ and ‘0’, respectively. By analyzing Figure 7, it can be deduced that the yellow graph has the maximal normalized intensity at output Q(t) and the minimal at output Q′(t); thus, the values assigned for ‘*a*’ and ‘*r*’ are 474 nm and 84.4 nm, with fabrication tolerances of ±20% (±9.48 nm) and ±9.2% (±7.8 nm), respectively.

**Figure 8:**
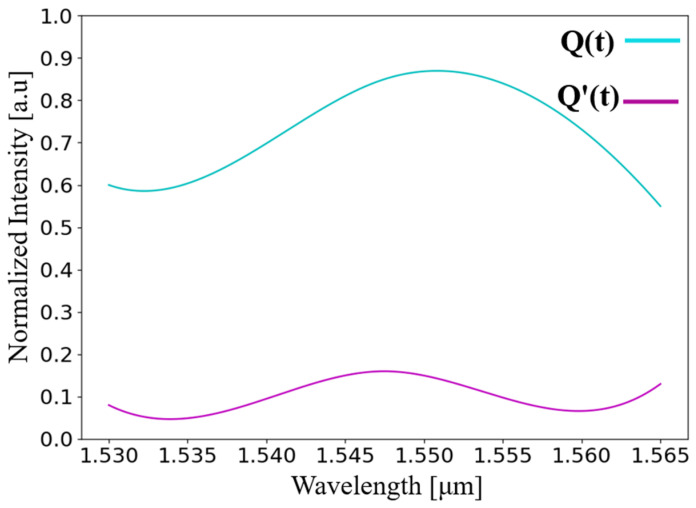
Normalized intensity at outputs Q(t) and Q′(t) as a function of input wavelength in the C-band spectrum.

Light sensitivity determines how stable a structure is when operating at non-optimal wavelengths. Light sensitivity is an important parameter. As in real-world conditions, there are imperfections and variations in input light waves. Optimization was performed, as illustrated in Figure 8, on the normalized intensity at the outputs Q(t) and Q′(t), with respect to the wavelength within the C-band spectrum, 1530–1565 nm.

From Figure 8, it is deduced that for all the wavelengths in the C-band spectrum, the normalized power at Q(t) output is above 0.6 a.u, and the normalized power at Q′(t) output is below 0.2 a.u. It can also be seen that the optimal wavelength is 1550 nm, and the device is operable with ±1% (±15.5 nm) of variation in the wavelength. These results indicate that the device has high stability and is reliable for imperfect inputs.

**Figure 9:**
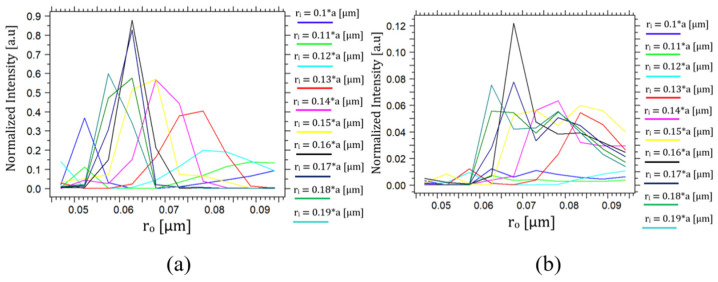
Normalized intensity at outputs (**a**) Q(t) and (**b**) Q′(t) as a function of ‘*r_i_*’ and ‘*r_o_*’.

The RR component consists of an inner ring made of Si rods with a radius ‘*r_i_*’ and an outer ring made of Si rods with a radius ‘*r_o_*’; the radius of these rods is critical for the optimal operation of the D-FF, as the radius affects the interferences of the light waves by changing the geometry of the component and, thus, the lengths that the light waves travel. The optimization of ‘*r_i_*’ and ‘*r_o_*’ was performed simultaneously, as shown in Figure 9a,b. The normalized intensity at the outputs Q(t) and Q′(t) as a function of ‘*r_i_*’ and ‘*r_o_*’ was in the range of 0.1*a* to 0.19*a*, when ‘*a*’ is the lattice constant that was optimized to be 474 nm.

Figure 9 reveals that the black curve exhibits the highest normalized intensity at output Q(t) and the lowest at output Q′(t). Accordingly, the values designated for *r_i_* and *r_o_* are 0.16*a* and 0.13*a*, respectively, where ‘*a*’ represents the optimized lattice constant set at 0.474 μm. The fabrication tolerances for ‘*r_i_*’ and ‘*r_o_*’ are ±18.75% (±14.22 nm) and ±19.35% (±12 nm).

**Figure 10:**
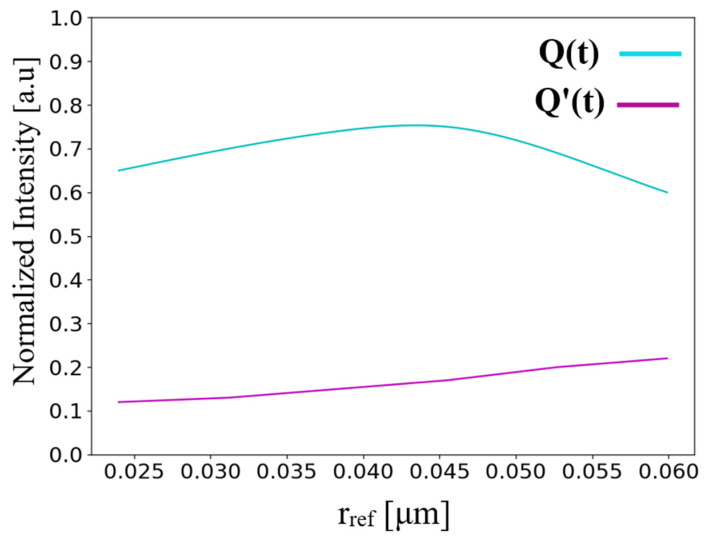
Normalized intensity at the outputs Q(t) and Q′(t) as a function of ‘*r_ref_*’.

The reflection rod’s radius ‘*r_ref_*’ impacts the traveling light waves and the power losses at the output of the device. Figure 10 visualizes the normalized intensity at the outputs Q(t) and Q′(t) as a function of the reflection rods in the range of 0.3*r* to 0.7*r*. This is when ‘*r*’ is the radius of the regular rods and was optimized to be 84.4 nm.

Figure 10 indicates that the optimal value for *r_ref_* is 0.5r, where ‘*r*’ represents the radius of the regular rods and has been fine-tuned to be 84.4 nm with a fabrication tolerance of ±30% (±18 nm).

## 5. Simulation Results

The PWE principle was utilized for solving the PBG. The FDTD method was employed to simulate the transmission diagram and reflectance of optical power in the output of the D-FF by inserting a Gaussian pulse at the inputs with different logic states.

**Figure 11:**
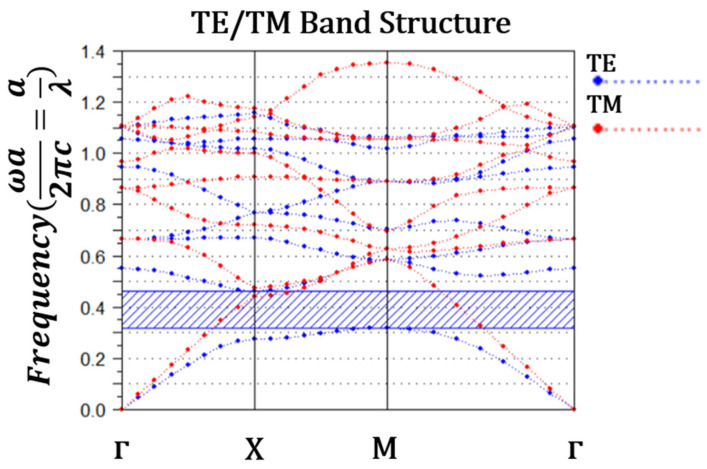
Band diagram of all-optical D-FF.

Figure 11 was generated using the Band-Solve tool, illustrating the TE polarization mode PBG for the proposed D-FF.

The bend diagram illustrates that the PBG is evident only in the TE polarization mode, aligning with expectations based on the distinctive configuration of the structure and occurs in the range of $0.25\leq \frac{a}{\lambda }\leq 0.36$. Where ‘*a*’ denotes the lattice constant, set at 474 nm, this allows us to deduce the range of compatible wavelengths for our design to be 1299 nm≤λ≤1887 nm. Thus, our design is compatible with the C-band spectrum and was optimized to work at 1550 nm.

**Figure 12:**
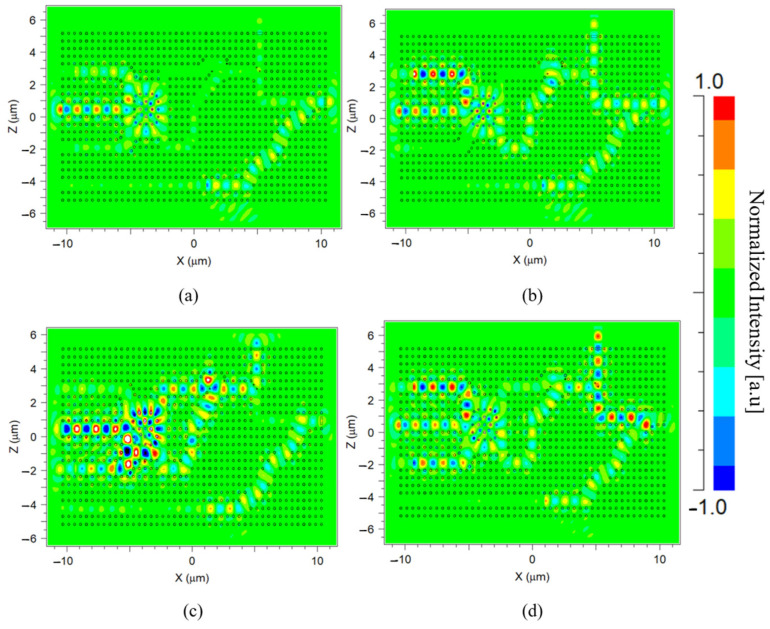
Light propagation diagram for input logic states (Clk, Q(t − 1), D): (**a**) 0, 0, 0, (**b**) 0, 0, 1, (**c**) 0, 1, 0, and (**d**) 0, 1, 1.

**Figure 13:**
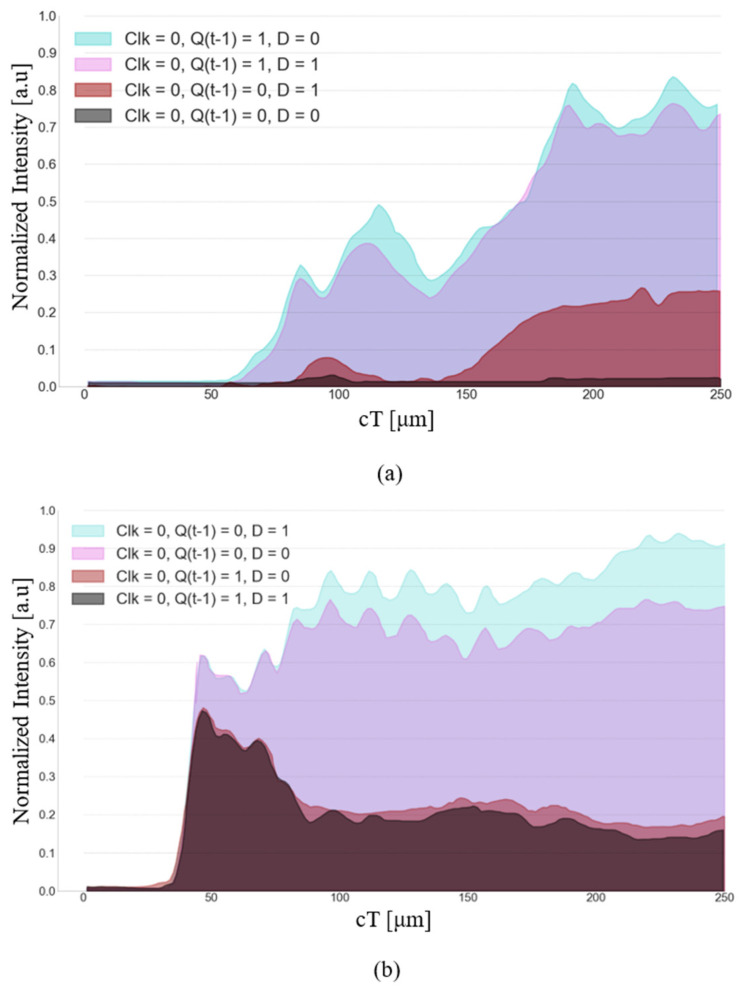
Normalized intensity at the outputs: (**a**) Q(t) and (**b**) Q′(t), where Clk = 0.

**Figure 14:**
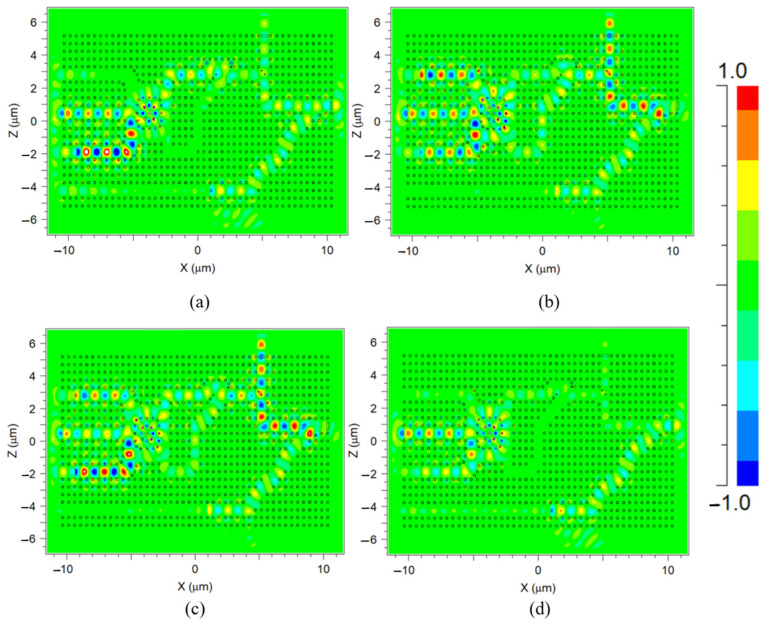
Light propagation diagram for input logic states (Clk, Q(t − 1), D): (**a**) 1, 0, 0, (**b**) 1, 0, 1, (**c**) 1, 1, 0, and (**d**) 1, 1, 1.

**Figure 15:**
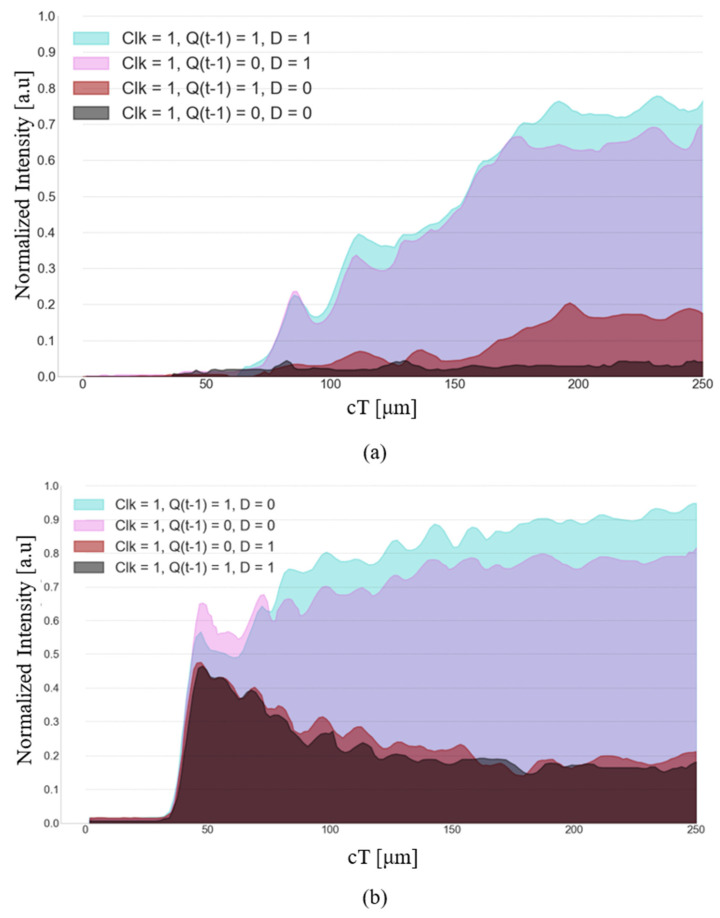
Normalized intensity at the outputs: (**a**) Q(t) and (**b**) Q′(t), where Clk = 1.

The performance of the suggested D-FF structure was simulated using the FDTD method. Four out of the eight results are presented in Figure 12a–d, each subfigure depicts a distinct logic state where Clk is logic ‘0’. Figure 13a,b depict the normalized intensity at the outputs Q(t) and Q′(t) for the same logic states. Figure 14a–d and Figure 15a,b depict the performance of the structure and the normalized intensity at outputs Q(t) and Q′(t), respectively, for the remaining four logic states where Clk is logic ‘1’.

Figure 13 illustrates the normalized intensity at outputs Q(t) and Q′(t) for the four input logic states with Clk set to logic ‘0’. The Y-axis represents the normalized intensity of the signal at each output, and the X-axis ‘cT’ is the time times the speed of light.

For the logic state (0, 0, 0), the normalized intensity at outputs Q(t) and Q′(t) is 0.02 a.u and 0.7 a.u, respectively, at the point cT = 150 μm. For the logic state (0, 0, 1), the normalized intensity at outputs Q(t) and Q′(t) is 0.22 a.u and 0.85 a.u, respectively, at the point cT = 200 μm. For the logic state (0, 1, 0), the normalized intensity at outputs Q(t) and Q′(t) is 0.75 a.u and 0.18 a.u, respectively, at the point cT = 200 μm. For the logic state (0, 1, 1), the normalized intensity at outputs Q(t) and Q′(t) is 0.72 a.u and 0.15 a.u, respectively, at the point cT = 200 μm. A summary of the results is shown in Table 4.

Figure 15 illustrates the normalized intensity at outputs Q(t) and Q′(t) for the four input logic states with Clk set to logic ‘1’.

Specifically, for the logic states (1, 0, 0), the normalized intensity at outputs Q(t) and Q′(t) is 0.02 a.u and 0.75 a.u, respectively, at the point cT = 150 μm. For the logic state (1, 0, 1), the normalized intensity at outputs Q(t) and Q′(t) is 0.65 a.u and 0.15 a.u, respectively, at the point cT = 150 μm. For the logic state (1, 1, 0), the normalized intensity at outputs Q(t) and Q′(t) is 0.15 a.u and 0.9 a.u, respectively, at the point cT = 200 μm. For the logic state (1, 1, 1), the normalized intensity at outputs Q(t) and Q′(t) is 0.72 a.u and 0.15 a.u, respectively, at the point cT = 190 μm. A summary of the results is shown in Table 4.

Upon inspecting each simulation result individually, it becomes apparent that the light intensity at the outputs becomes steady after a certain period known as stabilized intensity. The duration required to reach this stabilized intensity is referred to as the stabilization time. In the case of the proposed D-FF structure, the stabilization time is determined by the longest duration taken to achieve the stabilized intensity, resulting in a stabilization time of 0.66 picoseconds. Furthermore, the logic states ‘0’ and ‘1’ can be characterized by the normalized intensity falling within the ranges of 0 a.u to 0.2 a.u and 0.6 a.u to 1 a.u, respectively. The intensity range for logic state ‘0’ is defined as the maximum allowable intensity considered as logic ‘0’, and a similar principle is applied to the intensity range for logic state ‘1’, where the range is determined by the minimum acceptable intensity considered as logic ‘1’. The CR is computed using Eq. [1], reflecting the transmission losses, and is determined to be 4.77 dB.

A comparison between the proposed all-optical D-FF structure and other structures previously proposed in published papers is shown in Table 5. The main characteristics of the D-FF are the stabilization time, CR, footprint, and operating wavelength. All structures compared are composed of linear, square lattice PC structures. Table 5 demonstrates that the proposed D-FF structure presents a compelling blend of rapid stabilization time, competitive contrast ratio, optimized footprint, and compatibility with standard operating wavelengths. These attributes position it as a promising contender for advancing the realm of all-optical signal processing and logic operations. Notably, the primary breakthrough lies in the utilization of a silica substrate in the proposed photonic D-FF structure, diverging from conventional air substrates. This innovative shift enhances fabrication compatibility with CMOS processes, streamlines production, and bolsters scalability, marking a significant stride forward in the field of photonic all-optical signal processing and logic operations.

## 6. Conclusions

The proposed structure has been validated through simulations to effectively function as a classical D Flip Flop. This stands in contrast to other studies referenced in Table 5, which have demonstrated four logic states out of eight. This discrepancy arises from their oversight in acknowledging that the output must directly reflect the last value it retained, a fundamental principle enabling the component to retain memory. Another advantage of the proposed structure, distinguishing it from previous studies, lies in the innovative use of SiO_2_ as a substrate and as silicon rods. This approach enables fabrication and physical testing for the first time, while also offering the potential for hybridization with CMOS technology. This compatibility with CMOS technology opens up new avenues for integration and scalability, further enhancing the versatility and applicability of the proposed structure. In contrast, prior research primarily focused on air as the substrate.

The proposed structure is engineered to operate within the C-band, exhibiting low sensitivity to changes in wavelength and optimized performance at 1550 nm. It maintains operability within a ±1% variation in wavelength (1550 ± 15.5 nm), thereby expanding the range of potential applications for the device. The logic states ‘0’ and ‘1’ are delineated within the ranges of 0 a.u to 0.2 a.u and 0.6 a.u to 1 a.u, respectively, with a CR of 4.77 dB. The device achieves an overall stabilization time of 0.66 picoseconds and has a footprint of 21 μm × 12 μm.

Our D-Flip-Flop design displays impressive fabrication tolerances. With radii fabrication tolerances ranging from a minimum of 7.8 nm to a maximum of 18 nm, along with a pitch fabrication tolerance of 9.48 nm, our design achieves notable levels of precision. These tolerances ensure uniformity and reliability throughout the construction of each component, resulting in optimal performance under diverse operating conditions.

The device shows a clear distinction between logic ‘0’ and ‘1’, minimizing logic errors at the photonic decision circuit, and is a great advancement towards all-optical computing and memory units. Another promising use case for this design is a memory component for quantum computers. By observing the normalized intensity levels at the outputs, it is possible to set new logic states; for example, instead of just ‘0’ and ‘1’, power levels between 0.6 a.u and 0.8 a.u can be viewed as a new logic state.

The proposed design in this paper has an input that acts as the previous value at the output. A proposition for future research is connecting output Q(t) to input Q(t − 1) through a waveguide similar to those used in this paper, thus creating a dependency between the logic states and time. Essentially, the distance that the light will travel in the proposed waveguide will determine the time that the D-FF will hold a value and act as a memory component.

The proposed photonic D-FF presents a significant advancement towards establishing photonic arithmetic logic units. Its ability to effectively function within the C-band, low sensitivity to wavelength variations, and impressive fabrication tolerances make it a crucial component for all-optical computing architectures. Furthermore, its potential integration into quantum computing frameworks, leveraging its distinct logic states and memory capabilities, marks a promising pathway towards realizing advanced quantum computing systems.

## Figures and Tables

**Table 1:** Logic states of RR component.

D			Clk			Q(t − 1)			Output1		Output2	
N.I [a.u]	Phase Shift	Logic	N.I [a.u]	Phase Shift	Logic	N.I [a.u]	Phase Shift	Logic	N.I [a.u]	Logic	N.I [a.u]	Logic
0.1	0	0	0.9	π	0	0.1	π	0	0.08	0	0.05	0
0.1	0	0	0.9	π	0	0.9	π	1	0.7	1	0.03	0
0.1	0	0	0.9	0	1	0.1	π	0	0.05	0	0.08	0
0.1	0	0	0.9	0	1	0.9	π	1	0.15	0	0.3	0
0.9	0	1	0.9	π	0	0.1	π	0	0.3	0	0.17	0
0.9	0	1	0.9	π	0	0.9	π	1	0.3	0	0.8	1
0.9	0	1	0.9	0	1	0.1	π	0	0.7	1	0.05	0
0.9	0	1	0.9	0	1	0.9	π	1	0.8	1	0.33	0

**Table 2:** Logic states of T-splitter and OR logic gate component.

Input1		Input2		Output1		Output2	
N.I [a.u]	Logic	N.I [a.u]	Logic	N.I [a.u]	Logic	N.I [a.u]	Logic
0.1	0	0.1	0	0.15	0	0.13	0
0.1	0	0.9	1	0.98	1	0.85	1
0.9	1	0.1	0	0.95	1	0.8	1
0.9	1	0.9	1	0.99	1	0.97	1

**Table 3:** Logic states of NOT logic gate component.

Input1		CI		Output1		Output2	
N.I [a.u]	Logic	N.I [a.u]	Logic	N.I [a.u]	Logic	N.I [a.u]	Logic
0.1	0	0.1	0	0.15	0	0.13	0
0.1	0	0.9	1	0.98	1	0.85	1
0.9	1	0.1	0	0.95	1	0.8	1
0.9	1	0.9	1	0.99	1	0.97	1

**Table 4:** Logic states of proposed D-FF structure.

Input Clk			Input Q(t − 1)			Input D			Output Q(t)		Output Q′(t)	
N.I [a.u]	Phase Shift	Logic State	N.I [a.u]	Phase Shift	Logic State	N.I [a.u]	Phase Shift	Logic State	N.I [a.u]	Logic State	N.I [a.u]	Logic State
0.9	π	0	0.1	π	0	0.1	0	0	0.02	0	0.7	1
0.9	π	0	0.1	π	0	0.9	0	1	0.22	0	0.85	1
0.9	π	0	0.9	π	1	0.1	0	0	0.75	1	0.15	0
0.9	π	0	0.9	π	1	0.9	0	1	0.72	1	0.15	0
0.9	0	1	0.1	π	0	0.1	0	0	0.02	0	0.75	1
0.9	0	1	0.1	π	0	0.9	0	1	0.65	1	0.15	0
0.9	0	1	0.9	π	1	0.1	0	0	0.15	0	0.9	1
0.9	0	1	0.9	π	1	0.9	0	1	0.72	1	0.15	0

**Table 5:** Comparison of the proposed structure with similar works.

Structure Composition	Mechanism	Response Time [psec]	Contrast Ratio [dB]	Footprint [μm^2^]	Operating Wavelength [nm]	Year of Publication
Si rods in air [27]	Ring resonator, coupling rods, and scattering rods	0.063	11.13	71.14	1550	2020
Si rods in air [28]	MMI, edge rod point defects	0.29	9.63	71.28	1550	2017
Si rods in air [38]	Ring resonator with scattering rods	0.21	6.91	115.2	1552	2019
Si rods in air [39]	T-shaped waveguides	-	13.5	15.36	1550	2020
This paper: Si rods in SiO_2_	Ring resonator, OR and NOT logic gates, and T-splitter	0.66	4.77	252	1550	2024

## Data Availability

Data are contained within the article.
